# Obstructive Sleep Apnea and Cognitive Decline: A Review of Potential Vulnerability and Protective Factors

**DOI:** 10.3390/brainsci11060706

**Published:** 2021-05-27

**Authors:** Julie Legault, Cynthia Thompson, Marie-Ève Martineau-Dussault, Claire André, Andrée-Ann Baril, Guillermo Martinez Villar, Julie Carrier, Nadia Gosselin

**Affiliations:** 1 Center for Advanced Research in Sleep Medicine, Hôpital du Sacré-Coeur de Montréal, Recherche CIUSSS NIM, Montreal, QC H4J 1C5, Canada; julie.legault@umontreal.ca (J.L.); cynthia.thompson.cnmtl@ssss.gouv.qc.ca (C.T.); marie-eve.martineau-dussault@umontreal.ca (M.-È.M.-D.); claire.andre@umontreal.ca (C.A.); guillermo.martinez.villar@umontreal.ca (G.M.V.); julie.carrier.1@umontreal.ca (J.C.); 2 Department of Psychology, Université de Montréal, Montreal, QC H3C 3J7, Canada; 3 Douglas Mental Health University Institute, McGill University, Montreal, QC H4H 1R3, Canada; andree-ann.baril@umontreal.ca

**Keywords:** aging, sleep, obstructive sleep apnea, cognitive decline, mild cognitive impairment, Alzheimer’s disease, dementia, *ApoE* genotype, risk factors, comorbidities

## Abstract

Around 40% of dementia risk is attributable to modifiable risk factors such as physical inactivity, hypertension, diabetes and obesity. Recently, sleep disorders, including obstructive sleep apnea (OSA), have also been considered among these factors. However, despite several epidemiological studies investigating the link between OSA and cognitive decline, there is still no consensus on whether OSA increases the risk of dementia or not. Part of the heterogeneity observed in previous studies might be related to some individual characteristics that modulate the association between OSA and cognitive decline. In this narrative review, we present these individual characteristics, namely, age, sex, menopause, obesity, diabetes mellitus, hypertension, cardiovascular diseases, smoking, excessive alcohol consumption, depression, air pollution, *Apolipoprotein E ε4* allele, physical activity, and cognitive reserve. To date, large cohort studies of OSA and cognitive decline tended to statistically control for the effects of these variables, but whether they interact with OSA to predict cognitive decline remains to be elucidated. Being able to better predict who is at risk of cognitive decline when they have OSA would improve clinical management and treatment decisions, particularly when patients present relatively mild OSA.

## 1. Introduction

More and more people are living with dementia worldwide, with no curative treatment in sight. Alzheimer’s disease (AD) is by far the most common form of dementia, representing 60–70% of all dementia cases [1], and is generally preceded by a predementia stage of mild cognitive impairment (MCI). Around 40% of worldwide dementia cases are thought to be attributable to potentially modifiable risk factors, including diabetes, hypertension, obesity, physical inactivity, depression, smoking, low educational attainment, hearing impairment, low social contact, excessive alcohol consumption, and air pollution [2]. In addition, there is a growing body of evidence supporting the role of sleep disorders in the development of MCI and dementia [3,4,5], among which obstructive sleep apnea (OSA) could represent a modifiable risk factor of particular interest [6,7,8,9]. Therapeutic interventions targeting these modifiable risk factors may have the potential to delay dementia onset and slow its progression. Therefore, it is essential to properly identify these modifiable risk factors, and to fully understand the mechanisms by which they may increase the risk of dementia. Moreover, it is also essential to better understand how modifiable risk and protective factors interact to increase or reduce a person’s vulnerability to dementia. Better identifying individuals who could benefit from potential preventive therapies and those who should be included in clinical trials is a worldwide objective [10,11].

In this narrative review, we aim to identify individual vulnerability and protective characteristics that could have an impact on the relationship between OSA and cognitive decline, specifically MCI and AD. After defining OSA, we will present the potential mechanisms underlying the link between OSA and cognitive decline, and synthesize epidemiological studies investigating this association. We will then discuss potential vulnerability and protective characteristics that may moderate the association between OSA and cognitive decline, namely, age, sex, menopause, obesity, diabetes mellitus, hypertension, cardiovascular diseases, smoking, excessive alcohol consumption, depression, air pollution, *Apolipoprotein E ε4* (*ApoE4*) allele, physical activity, and cognitive reserve. Understanding the roles and mechanisms of both vulnerability and protective individual characteristics could shed light on the discrepancies observed between cohort studies and eventually help individualize therapeutical intervention to prevent cognitive decline in OSA patients.

## 2. Definition and Prevalence of OSA

OSA is a respiratory disorder characterized by repeated episodes of partial (hypopnea) and complete (apnea) obstructions of the upper airway during sleep [12,13]. These recurrent respiratory events lead to cortical arousal and sleep fragmentation, intermittent hypoxemia, and increased sympathetic activity, affecting sleep quality and daytime functioning [14]. OSA severity is typically assessed with the apnea–hypopnea index (AHI), which is the average number of apneas and hypopneas per hour of sleep. The severity of OSA is determined according to the following thresholds: mild (AHI ≥ 5 and <15), moderate (AHI ≥ 15 and <30) or severe (AHI ≥ 30) [15]. According to a systematic review in the adult population, 9–38% of individuals present with mild OSA and 6–17% present with moderate to severe OSA [16]. In the elderly, these estimations reach up to 84% for mild OSA and 36% for moderate to severe OSA [16]. Even more worrying, one study observed that 56% of adults aged 65 and older were at high risk of OSA, while only 8% of them had been tested for OSA and diagnosed [17]. Thus, many older adults remain undiagnosed, and therefore not treated. Furthermore, when people do get diagnosed, only about 41% remain adherent to their continuous positive airway pressure (CPAP) treatment after one year [18]. This low CPAP adherence is alarming, especially considering that this treatment could delay cognitive decline in individuals with OSA [9].

## 3. Potential Mechanisms Linking OSA and Cognitive Decline in Older Adults

It is well established that OSA causes sleep fragmentation and intermittent hypoxemia. More specifically, repeated micro-arousals alter both sleep macroarchitecture (time spent in stage N3 and rapid-eye movement (REM) sleep) [19] and microstructure (slow-wave and spindle characteristics) [20,21,22,23]. Considering the critical roles of sleep continuity, slow-wave sleep, REM sleep, and sleep spindles in neurogenesis, brain plasticity, alertness, and memory formation and consolidation [24,25], chronic sleep changes caused by OSA could negatively affect cognitive health [6,26,27]. REM-dependent OSA could be particularly harmful to the brain, with respiratory events occurring during this sleep stage being associated with reduced daytime regional cerebral blood flow, even in mild OSA [28]. Indeed, muscle atonia during REM sleep can increase the occurrence and the hypoxic levels of respiratory obstructive events, and thus, some individuals present respiratory events mostly in this sleep stage. Further, REM-dependent OSA is more strongly associated with excessive daytime sleepiness than NREM-OSA [29], which is in turn related to cognitive impairment [30].

Moreover, both sleep fragmentation and intermittent hypoxia interfere with brain structure and function, increasing their vulnerability to neurodegenerative diseases. Indeed, it has been suggested that a biphasic pattern of neuroimaging findings could be in play in OSA [31], with acute transitory or compensatory responses (i.e., gray matter hypertrophy, restricted white matter diffusivities) followed by evidence of cellular damage (i.e., gray matter atrophy, higher white matter hyperintensity burden, lower white matter fractional anisotropy, higher water diffusivities). In addition, OSA has been recently associated with increased amyloid and tau burden [21,32,33,34,35,36,37,38,39,40,41,42,43], two proteins involved in AD pathophysiology. Several mechanisms likely underlie these neuroimaging or pathological findings and include inflammation, oxidative stress, metabolic disturbances, cerebral edema and endothelial dysfunction [27]. Indeed, inflammation is involved in neurodegenerative processes, notably by triggering a positive feedback loop that increases amyloid beta production and oxidative stress, facilitating amyloid and tau pathology [26,44].

## 4. Cohort Studies Investigating the Association between OSA and Cognitive Decline in Older Adults

### 4.1. Cross-Sectional Studies

The majority of large cross-sectional cohort studies investigating the association between OSA and cognitive impairment in middle-aged and older adults have used objective sleep measures, i.e., polysomnography or portable devices, to evaluate OSA [45,46,47,48,49,50,51,52], and one study used a questionnaire to screen for OSA [53]. While some studies have investigated cognitive performance using comprehensive neuropsychological batteries [51,53,54], others used a limited number of neuropsychological tests targeting specific cognitive functions or global functioning [45,46,49,52]. In studies showing that OSA is associated with poorer cognitive functioning, associations were found for long-term verbal memory [47], working memory [47] and global cognition [52]. The OSA severity markers or OSA-related symptoms associated with cognition were highly heterogeneous across studies, including snoring [53], self-reported apneas [53], hypoxemia [45,54] and AHI [50,51]. Other studies have not found a significant association between any OSA severity marker and cognition [46,48,49].

Despite the heterogenous results emerging from cross-sectional studies, associations between OSA and cognitive functioning seem better established in the domains of attention, memory, and processing speed, while less evidence supports an association between OSA and working memory, executive functions, language and visual abilities in middle-aged and older adults. Interestingly, in younger adults, the most affected cognitive domains in OSA are attention, episodic memory, working memory, and executive functions [14]. This suggests that while some cognitive domains appear to be affected by OSA regardless of the age (namely, memory and attention), other cognitive domains seem less impacted by OSA with increasing age, such as executive function and working memory.

### 4.2. Longitudinal Studies

Longitudinal cohort studies have the advantage of quantifying cognitive decline over time. They used self-reported diagnoses (e.g., [9,55]) or in-home polysomnography or portable devices (e.g., [7,56,57,58,59]) to identify OSA cases. Furthermore, the majority of these studies used global cognitive measures or screening tools, such as the Mini-Mental State examination (MMSE; e.g., [7,59]), while a comprehensive neuropsychological battery was used in one study [58].

Among the main longitudinal cohort studies, the Study of Osteoporotic Fractures included 298 82-year-old women and found that 45% of women with OSA developed MCI or dementia at five-year follow-up, compared to 31% of women without OSA [59]. However, neither of the two neuropsychological tests used to assess global cognition and executive functions could identify impairment of specific cognitive functions related to OSA. This result was also obtained in the Atherosclerosis Risk in Communities Study that included men and women aged 45–64 years and failed to show an association between OSA and specific cognitive tests at 15-year follow-up [56], while highlighting an increased risk of dementia in individuals with severe OSA [57]. In the Osteoporotic Fractures in Men Sleep Study, including men aged 65 and older, another team showed a negative relationship between baseline nocturnal hypoxemia and global cognitive functioning after three years, while executive functions were not associated with OSA [7]. On the other hand, the Proof-SYNAPSE study used a wide selection of neuropsychological tests in men and women aged 65 years and showed only a slight decline in attention related to OSA after eight years, without any change in executive functions and memory [58].

In summary, studies using measures of global cognition and clinical diagnosis of cognitive impairment or dementia were more susceptible to highlight cognitive decline associated with OSA over time than those using comprehensive neuropsychological batteries [56,57,58]. This suggests that longitudinal studies are more susceptible to identify major OSA-related cognitive decline over time, but not milder cognitive decline in specific domains.

### 4.3. Meta-Analyses, Meta-Reviews and Systematic Reviews

Most meta-analyses and systematic reviews concluded that a significant association between OSA and cognition exists [8,60,61,62] and that OSA increases the risk of AD [63,64,65]. Interestingly, these meta-analyses and systematic reviews highlighted the fact that small cohorts and controlled case studies from sleep clinics have shown effects of OSA on cognition [66,67], while most studies of large community-based cohort studies failed to show significant associations [45,56]. Another systematic review concluded that the link between OSA and cognition is weak [68], possibly due to the age range used (50 years and over, while others included studies of participants aged 30 years and over [8,60,62]). Similarly, another systematic review showed a significant association between OSA and cognition only in adults younger than 60 years [60]. In addition to age, the variability in the strength of the association could also be due to the study types and designs, the recruitment methods and/or that only more severe OSA cases are associated with cognitive dysfunction.

## 5. Vulnerability Risk Factors

Not all adults with OSA are at high risk of developing MCI or dementia. Individual characteristics are likely to add to or interact with OSA severity to explain the increased risk of cognitive decline when present. Some variables are difficult to quantify, such as OSA disease duration, and will not be discussed here. Rather, we will focus on factors that should be included as moderating factors in future studies investigating the risk of cognitive decline in OSA. Of note, some studies considered potential moderating variables as confounders, such as *ApoE4*, sex and age. These are discussed in detail in the following subsections. Table 1 summarizes these potentially moderating factors.

**Table 1:** Individual characteristics that could interact with OSA severity to predict risk of cognitive decline.

Evidence Availability	Individual Characteristics	Individuals with OSA Potentially at Increased Risk of Cognitive Decline	Studies Supporting the Potential Role
A few studies have tested these variables	Sex	Women	[52,69,70,71]
	*ApoE4*	*ApoE4* carriers	[47,52,72,73]
Almost no studies have tested these variables	Menopause	Postmenopausal women	[74]
	Smoking	Smokers	[75]
	Cognitive reserve	Individuals with a low cognitive reserve	[76]
Heterogeneous results or not tested	Age	Young and middle-aged adults	[60]
	Obesity	Obese individuals	[26,77]
	Diabetes mellitus	Diabetic individuals	No evidence found
	Hypertension	Individuals with hypertension	[78,79]
	Cardiovascular diseases	Individuals with cardiovascular diseases	No evidence found
	Excessive alcohol consumption	Individuals having moderate to high alcohol consumption	No evidence found
	Depression	Individual with depression	[80]
	Physical activity	Inactive individuals	[81]
	Air pollution	Individuals exposed to high levels of air pollution	No evidence found

### 5.1. Age

Age is a well-established risk factor for both OSA [17,82] and dementia [83]. The increased prevalence of OSA with age could be explained in part by indirect factors that are associated with dementia risk (e.g., increasing body mass index, menopause, increasing prevalence of health comorbidities), but also by the marked decrease in tongue and palate muscle activity during sleep in older adults [84,85]. We could therefore hypothesize that older age potentiates the negative effects of OSA on brain health, but the opposite seems to be observed. In fact, a recent systematic review that included 68 studies showed that young and middle-aged adults (30 to 60 years old) suffering from OSA had impairments in attention, executive functions and memory, while apneic adults aged 60 and over had cognitive functioning comparable to non-apneic people of the same age [60]. It is possible that other conditions occurring mainly with advancing age, such as cardiovascular diseases, hypertension and neurodegenerative diseases, could significantly influence cognitive functioning in the elderly, and therefore hide or blur the association between apnea and cognition. This could explain the weaker association between OSA and cognition; however, this hypothesis remains to be tested.

### 5.2. Sex and Menopause

The prevalence of dementia is higher in women by 19–29% in many parts of the world, including Europe, Latin America, Australia, and areas outside of the Pacific region of Asia [86], and women represent approximately two-thirds of AD dementia cases in the United States [87]. In addition to women’s longer life expectancies as compared to men’s, this disparity could be due to the different effects of some risk factors in men and women, with some being more common (e.g., lower access to education) and having a stronger impact in women than in men (e.g., *ApoE4* genotype), and others being specific to women (e.g., menopause) [87].

With respect to OSA, men, as compared to women, tend to accumulate more fatty tissue in the upper body, especially in the pharynx, which promotes airway collapse and blockage leading to OSA [12]. Accordingly, epidemiological studies using polysomnography, questionnaires or self-reported OSA diagnoses found a prevalence ranging from 13% to 31% in men and 4% to 21% in women [88,89,90,91,92,93]. Moreover, men and women with OSA seem to experience different symptoms. Men report snoring, shortness of breath and apnea observed by the bed partner, while women report non-specific symptoms such as headaches, fatigue, depression, anxiety and insomnia [94,95], suggesting a sex-specific impact of OSA on diurnal functioning. Of note, these sex differences might reflect an assessment bias. In fact, since initial research was mainly conducted in men, their symptoms appear to be more typical, while women’s symptoms might be as frequent, but have received less attention. Furthermore, women might experience similar symptoms as men, but these symptoms might be less frequently reported by their bed partners.

Few studies have examined sex differences in the association between OSA and cognition. Among them, two Taiwanese studies found an interaction effect between sex and sleep disturbances on cognition [71,96]. Chiu et al. [96] showed that men reporting difficulty breathing during sleep, habitual snoring, and prolonged sleep duration (>8.5 h) were at higher risk of cognitive impairment, whereas in women, only prolonged sleep duration was associated with cognitive impairment. However, Chang et al. [71] showed that women with OSA, but not men, were more likely to develop dementia. Similarly, the Hispanic Community Health Study showed no association between OSA and cognition in men, while a high AHI in women was associated with a marked decrease in cognitive performance in all three cognitive domains assessed (executive function, memory and information processing speed) [69]. Age stratified analyses indicated that this sex effect was specific to the 45–54 age group, which corresponds to the age of perimenopause in most women. In older women (65–74 years), only an association between OSA and information processing speed was observed. Interestingly, cross-sectional studies of cohorts including only women showed significant associations between OSA and poorer cognition [52,70], while cohorts including only men showed no such association [45,49]. Neuroimaging studies from one group included between 10 and 16 women with OSA and highlighted OSA and sex interactions, where only women showed white matter alterations [97], cortical thinning [98] and unilateral volume changes in the hippocampus [99].

Physiological mechanisms that could explain women’s vulnerability are still not clear, but the inflammatory response to OSA is among those suspected. An interesting study of apneic adults without known comorbidities showed an increase in inflammatory biomarkers and cardiovascular risk markers (e.g., C-reactive protein, fibrinogen and elevated erythrocyte sedimentation rate) particularly in women, even though apneic men showed higher levels of uric acid, a marker of hypoxia [100]. With equivalent AHI, women have higher levels of fibrinogen and C-reactive protein compared to men [101].

Menopause is another factor that could explain women’s vulnerability to OSA. Importantly, menopause is accompanied by decreased metabolic activity and increased amyloid burden, characteristics of an AD endophenotype [102]. In fact, a three-year longitudinal study observed postmenopausal women and showed a higher rate of amyloid deposition compared to pre-menopausal women and men, and a higher rate of hippocampal volume loss compared to pre- and perimenopausal women and men [103]. AD pathology appears years to decades prior to clinically detectable symptoms, corresponding to the age of perimenopause in most women [102]. Menopause transition could thus increase the risk of AD in women.

Menopause is characterized by estrogen and progesterone depletion, which triggers an adipose tissue distribution change, making this distribution similar to what is observed in men [13]. Postmenopausal women have more adipose accumulation in the upper body and increased body mass index (BMI), neck circumference, and waist-to-hip ratio, all increasing their risk of OSA [104]. Indeed, postmenopausal women not using hormone replacement therapy are three to four times more likely than non-menopausal women to suffer from OSA, suggesting a protective role for estrogen and progesterone against OSA [13,105].

Postmenopausal women at high risk of OSA, as assessed by the Berlin Questionnaire [106], reported more subjective cognitive impairment than those at low risk of OSA [74]. An interesting review about ovarian hormones, sleep, and cognition suggests that the loss of ovarian hormones following menopause could increase the development of sleep disorders, and thus could precipitate cognitive decline and dementia in women [107]. More precisely, increases in sleep disorders could enhance inflammation, leading to neurodegeneration and cognitive decline. Thus, menopause could accelerate OSA-related cognitive decline. Although limited data are available, a recent meta-analysis showed that hormone replacement therapy improves cognitive function in women with AD [108]. Since hormone replacement therapy improves sleep in some, but not all studies [107], its potential to reduce OSA-related vulnerability to cognitive decline needs to be investigated.

### 5.3. Obesity

Results from a 36-year longitudinal study showed that middle-aged adults with a combination of obesity and high abdominal circumference have a 3.6-fold increase in dementia risk, even after controlling for diabetes and other vascular comorbidities [109]. This increased risk can be explained by the fact that adipocytes secrete pro-inflammatory cytokines that alter synaptic and neuronal plasticity, which in turn contributes to neurodegenerative processes [110]. Furthermore, obesity-related inflammation leads to oxidative stress, which also plays a role in neurodegenerative processes [111].

The risk of OSA increases progressively with BMI and is even more strongly associated with neck circumference [112]. Obesity is the strongest risk factor for OSA and its relationship with OSA might be bidirectional [113]. While obesity is linked to several co-morbidities exacerbated by OSA, such as myocardial infarction, congestive heart failure, stroke, type 2 diabetes and hypertension, OSA is thought to increase the risk of obesity due to the physical inactivity associated with daytime sleepiness [113].

Considering obesity’s possible contribution to neurodegenerative processes and its strong link to OSA, Polsek et al. [26] suggested that co-morbid obesity could promote AD progression in individuals with OSA. Yet, most studies investigating the link between OSA and cognitive decline have statistically controlled for obesity but have not stratified their analyses according to obesity. Interestingly, one recent study found that obese OSA patients have reduced performance in working memory and psychomotor vigilance compared to non-obese OSA patients [77]. More studies are therefore needed to verify if overweight or obese adults are particularly vulnerable to cognitive alterations linked to OSA.

### 5.4. Diabetes Mellitus

A pooled meta-analysis of 2.3 million individuals with type 2 diabetes showed that diabetes is associated with a 1.6-fold increase in dementia risk [114]. In addition to a possible increase in amyloid and tau burden in diabetic patients [115,116], non-AD mechanisms could also link type 2 diabetes to neurodegeneration, as a result of insulin resistance disturbing cerebral insulin pathways, vascular endothelial dysfunction leading to hypoxic neuronal injury and inflammation disrupting the blood–brain barrier [117].

Up to 30% of patients with OSA suffer from type 2 diabetes [118], and up to 86% of obese patients with type 2 diabetes suffer from OSA [119]. The link between OSA and diabetes is believed to be bidirectional but has not yet been fully elucidated. On the one hand, the autonomic dysfunction characteristic of diabetes could lead to respiratory instabilities, increasing the risk of OSA [120]. On the other hand, intermittent hypoxemia, sleep fragmentation and reduced time spent in N3 and REM sleep in OSA may increase the risk of developing alterations in glucose metabolism such as insulin resistance, glucose intolerance and type 2 diabetes [120,121,122,123].

Regarding the interaction between diabetes and OSA, a study of adults (mean age = 55 years) with prediabetes or type 2 diabetes found no association between OSA and cognitive performance [124]. It is important to mention that most participants (43%) had only mild OSA in that study. Thus, more studies are needed to verify if cognitive impairment and decline are particularly important in diabetic individuals with mild, moderate or severe OSA.

### 5.5. Hypertension

Results from the Framingham Offspring cohort study showed that midlife systolic hypertension and the persistence of systolic hypertension into later life were associated with a 1.6 to 2-fold increase in dementia risk at 18-year follow-up [125]. Various pathways could link hypertension to dementia, including small vessel disease, large artery atherosclerosis, and hypertension-related cardiac dysfunction, which predispose to cerebral hypoperfusion [125].

An estimated 50% of patients with hypertension present with OSA [126]. Conversely, individuals with OSA have an extra 1.8-fold risk of resistant hypertension compared to those without OSA [127]. The link between OSA and hypertension could be explained by anatomical changes resulting from hypoxia. In fact, intermittent hypoxia triggers an intense cardiovascular response, leading to sympathetic overactivity, which in turn contributes to hypertension [128]. It is therefore possible that intermittent hypoxemia and sleep fragmentation accentuate the effects of hypertension on cognitive decline, while hypertension may in turn potentiate the effects of OSA on the risk of dementia by increasing oxidative stress and inflammatory response [78]. Moreover, a study of men and women aged 60 years and older showed working memory impairment related to OSA and hypertension [79].

### 5.6. Cardiovascular Diseases

Dementia and cardiovascular diseases share common risk factors that could lead to cognitive decline, such as diabetes mellitus, smoking, and hypertension [129]. Moreover, studies have also linked dementia to specific cardiovascular diseases, such as coronary heart disease, atrial fibrillation and heart failure [129]. While it is not yet established if cardiovascular diseases per se increase the risk of dementia or if it is due to shared risk factors, cardiovascular diseases could contribute directly to cognitive decline through cerebral hypoperfusion, hypoxia, embolisms, and infarcts [129]. Moreover, one study showed that genetic predisposition to coronary artery disease increases dementia risk three years after a cardiovascular disease diagnosis [130].

It is difficult to isolate the independent role that cardiovascular diseases may play in OSA because of the concomitant presence of other cardiovascular risk factors such as obesity and glucose intolerance [131]. However, the prevalence of OSA is estimated to be two to three times higher in people with cardiovascular diseases [132], and up to 50% of apneic patients show cardiac and metabolic abnormalities [131]. Fragmented sleep and recurrent cycles of decreased oxygen levels and reoxygenation associated with OSA lead to oxidative stress and inflammation, which damage blood vessel walls and increase hypertension [133,134]. Considering the cascade of deleterious consequences produced by both OSA and cardiovascular diseases, it is likely that treating OSA could help mitigate their negative impacts on brain health.

### 5.7. Smoking

It is highly recognized that smoking increases the risk of cognitive decline and dementia [2,135]. A multi-ethnic cohort study including adults aged 50–60 years indicates that smoking more than two packs of cigarettes daily doubles the risk of dementia at 23-year follow-up [136]. In addition, a Chinese study conducted with current, past and non-smokers aged 20–60 years who reported snoring or daytime drowsiness showed that the coexistence of OSA and chronic smoking results in more pronounced cognitive impairment than smoking alone [75]. Specifically, inhaling cigarette smoke increases oxidative stress and systemic inflammation, phenomena also observed in OSA [75]. Thus, the concomitant presence of smoking and OSA could precipitate cognitive decline.

### 5.8. Excessive Alcohol Consumption

A scoping review of 28 systematic reviews revealed that excessive alcohol consumption is linked to an increased dementia risk, while low to moderate drinking is associated with a decreased risk [137]. Alcohol could lead to brain damage directly, through its neurotoxic effect on brain structure and function [137]. In fact, chronic alcohol abuse could lead to loss of white matter, with astrocytes, oligodendrocytes, and synaptic terminals being particularly vulnerable to the toxic effects of alcohol [138]. Moreover, heavy alcohol consumption can cause high blood pressure, ischemic heart disease, cardiomyopathy, atrial fibrillation, and strokes, which are in turn associated with increased risk of vascular dementia [137,139]. Low to moderate alcohol consumption is associated with lower odds for dementia than abstainers; while this link is not yet well understood, the inclusion of former drinkers who might have already suffered from alcohol-related consequences in the group of abstainers could explain part of the results [140].

Due to its relaxing properties, alcohol increases upper airway collapsibility and could thus represent a risk factor for the occurrence of apneas when it is ingested before bedtime [141,142,143]. Results from systematic reviews and meta-analysis revealed that alcohol consumption is associated with a 25% increased risk of OSA [143], an increase in AHI of 2.33 to 3.98 events per hour, and a 0.60% to 2.72% decrease in lowest oxygen saturation [142,144]. Furthermore, results from the Wisconsin Sleep Cohort Study showed that alcohol’s negative impact on OSA may not be restricted to its consumption near bedtime, but to its habitual consumption [141]. In fact, for each increase of one self-reported drink per day, men had 25% greater odds of OSA, while women’s alcohol consumption was not associated with OSA [141]. However, no study has investigated the presence of cognitive dysfunction in OSA adults having moderate to high alcohol consumption. Thus, alcohol could represent a modifiable risk factor for dementia when consumed in excess and could also predispose to OSA even in a lower dosage.

### 5.9. Depression

Cohort studies and meta-analyses suggest that a history of depression is associated with an increased dementia risk, with depressive symptoms independently associated with cognitive decline [2,145]. However, it is not well established whether depression increases the risk of dementia or is an early marker of brain changes associated with dementia [2,135].

A systematic review and meta-analysis indicates that OSA is linked to depression, with longitudinal studies being more susceptible to highlight cognitive decline associated with OSA than cross-sectional studies [146]. In fact, two longitudinal studies suggested that OSA is an independent risk factor for depression, with participants with OSA being about twice as likely to be depressed as those without OSA [147,148]. According to a model developed by Kerner and Roose [80], cerebral hypoperfusion, endothelial dysfunction and neuroinflammation due to OSA could initiate or amplify the development of cerebral small vessel disease and blood–brain barrier dysfunction, resulting in white matter lesions, gray matter loss, white matter fiber tract abnormalities, neuronal damage, synaptic plasticity and neurodegenerative processes, in turn leading to depressive symptoms and cognitive impairment [80]. One study found that depressive symptoms observed in OSA patients were associated with accentuated excessive daytime sleepiness [149]. Whether depression in OSA patients is a daytime consequence of OSA, an early marker of neurodegeneration, or a risk factor that interacts with OSA to hasten cognitive decline is a highly relevant clinical question.

### 5.10. Air Pollution

Air pollution is a recognized risk factor for dementia [2,150,151]. A systematic review of longitudinal studies suggested that exposure to particulate matter, nitrogen dioxide and carbon monoxide increases dementia risk [151]. It has been hypothesized that the mechanisms underlying the effects of air pollution on brain health could include inflammation, microglial activation, reactive oxygen species, and the production and deposition of amyloid-beta peptides [150].

Although it is well known that air pollution has deleterious effects on respiratory health, its relationship with OSA remains unclear [152]. A recent systematic review including five cross-sectional studies of adults objectively assessed for OSA suggested a relationship between air pollution exposure and increased risk of OSA, with variability attributed to seasons, temperatures and geographic locations [152].

Furthermore, air pollution and OSA share common potential pathways to health conditions and cognitive decline such as hypertension, insulin resistance, oxidative stress, inflammation and endothelial dysfunction [151]. Laratta et al. [153] suggested an interaction of OSA and air pollution on systemic inflammation. In fact, this team observed increased levels of inflammation with high particulate matter exposure in individuals with suspected OSA, and with black carbon exposure in patients with moderate to severe OSA [153]. Considering the potential deleterious effects of both inflammation and health conditions on the brain, it is likely that OSA and exposure to air pollution have a joint negative effect on cognitive health. Moreover, air pollution and OSA could increase dementia risk through an indirect pathway related to metabolic dysfunctions such as diabetes [154], hyperglycemia and low high-density lipoprotein cholesterol [155]. Future studies should verify if exposure to air pollution makes patients particularly vulnerable to cognitive alterations linked to OSA.

### 5.11. ApoE4

ApoE is a protein involved in cholesterol transport, growth and repair of the nervous system during development or after injury, synaptic and dendritic remodeling, and the scavenging of amyloid [156,157,158]. Carriers of the *ApoE4* allele have decreased expression of the ApoE protein, and thus, its beneficial role in the central nervous system is reduced.

The *ApoE4* allele remains, to this day, the strongest genetic risk factor for sporadic AD [156,159]. Most studies have shown an association between *ApoE4* allele carrier status and poor performance in various cognitive domains in elderly people without dementia [160,161,162]. Even with the increased prevalence of OSA in older individuals, these studies did not take into account the presence or absence of OSA in their analyses. However, the presence of *ApoE4* alone is not sufficient to cause cognitive decline and is thought to be a susceptibility factor that interacts with other genetic and environmental influences to increase the risk of cognitive decline [162,163]; one of these factors could be OSA.

While one study showed that *ApoE4* was associated with an increased risk of moderate to severe OSA and higher AHI [164], others did not show this link [165,166,167]. A few studies have shown that in apneic patients aged 30 or over, the presence of the *ApoE4* allele is associated with more cognitive impairment as compared to non-carriers [47,52,72,73]. These results could be explained by the vulnerability of *ApoE4* carriers to central nervous system injuries of various origins, such as the oxidative stress associated with OSA [72,73]. Whether cognitive impairment in *ApoE4* carriers with OSA progresses to dementia is a question that must be investigated.

## 6. Protective Factors

### 6.1. Physical Activity

Physical inactivity is the most significant modifiable risk factor for AD in the United States, Europe and England, where approximately one-third of the adult population is physically inactive (i.e., less than 20 min of vigorous activity during three or more days or less than 30 min of moderate activity during five or more days per week) [168]. Results from randomized controlled trials in healthy inactive older adults show that low-intensity exercise programs improve cognitive functioning and decrease the risk of cognitive decline [135]. Physical activity promotes brain plasticity and neurogenesis as well as vascular health by reducing blood pressure, lipids, obesity, and markers of inflammation [169]. In addition, physical activity is associated with lower levels of AD pathology such as tau and beta-amyloid [170].

A systematic review of physical activity and OSA suggests that apneic patients often display low levels of physical activity, possibly due to the fatigue and daytime sleepiness characteristics of OSA [81]. Conversely, exercise training programs of at least a three-week duration are associated with a decrease in AHI and symptoms of drowsiness despite no change in BMI [81]. This decrease in OSA symptomatology may be due to increased upper airway dilator strength and fatigue resistance, decreased nasal resistance, and increased respiratory stability during deep sleep [171]. Physical activity could therefore improve OSA symptoms and cognitive performance, and possibly delay OSA-related cognitive impairment, but this needs to be tested.

### 6.2. Cognitive Reserve

The level of education, intelligence and main occupation are markers of the cognitive reserve, which makes it possible to withstand a deterioration of cerebral structures and to preserve cognitive and behavioral functions at an optimal level when a neurodegenerative process sets in [172]. Worldwide, low levels of education are believed to be the most important modifiable risk factor for AD [168].

With respect to OSA, higher intelligence is thought to have a protective effect against OSA-related cognitive decline, possibly due to the associated cognitive reserve, and may compensate for the hypoxic brain damage or daytime sleepiness associated with OSA [76]. In fact, one study showed similar levels of attention in highly intelligent participants with and without OSA, while in those with normal intelligence, apneic patients had reduced attention compared to the control group [76]. Since cognitive reserve is a well-established protective factor of dementia, future studies will need to determine whether cognitive reserve can delay, or even prevent, cognitive decline in apneic patients.

## 7. Clinical Impact and Future Directions

Clinicians often face a dilemma as to whether they should treat patients with mild OSA or those with low diurnal symptoms. When they prescribe OSA treatment, patients may refuse it or may fail to use it on an on-going basis. With the recent findings on OSA and the risk of cognitive decline, clinicians and patients should now take into account the risk of developing dementia if OSA remains untreated. Identifying vulnerability and protective characteristics in OSA and their impact on cognitive decline has the potential to guide clinicians in treatment decisions, for example, through the use of decision trees or software/web applications based on machine learning and available for clinicians. Patients presenting with OSA and multiple vulnerability factors for cognitive decline could thus be offered treatment even if they only have mild OSA. To improve the management of OSA in this at-risk population, systematic screening for OSA could be implemented in memory clinics and cardiology/cardiovascular hospital units.

Furthermore, studies investigating the link between OSA and cognitive decline often do not characterize participants with respect to underlying pathology. Since AD pathology is present during a long silent phase, it is possibly present in some participants and left undetected by cognitive screening. Since the influence of risk and protective factors possibly differs according to the presence or absence of AD pathology, future studies should quantify participants’ AD pathology. Finally, patients with multiple sleep disorders (i.e., those with OSA combined with insomnia, REM sleep behavior disorder, circadian misalignment) may be at higher risk of cognitive decline and dementia. The effects of having multiple sleep disorders should be investigated in future studies, rather than excluding patients based on comorbid sleep disorders.
